# Clinical efficacy of Bufei Yishen and Qutan Tongluo Cream on the treatment of patients with stable chronic obstructive pulmonary disease

**DOI:** 10.3389/fmed.2025.1472947

**Published:** 2025-04-16

**Authors:** Huicong Zhang, Youxia Zhou, Gang Song

**Affiliations:** ^1^Department of Respiratory, Hebei Provincial Hospital of Traditional Chinese Medicine, Shijiazhuang, China; ^2^Department of General Internal Medicine, Shijiazhuang Fourth Hospital, Shijiazhuang, China

**Keywords:** COPD, TCM, inflammation, acute exacerbation, hospitalization

## Abstract

**Background:**

Traditional Chinese medicine has been used for the treatment of chronic obstructive pulmonary disease (COPD). However, the effect of Bufei Yishen and Qutan Tongluo Cream (BYQTC) on the amelioration clinical symptoms of COPD remains unclear.

**Methods:**

The enrolled COPD patients were randomly divided into control and observation groups. The efficacy of BYQTC was assessed by the reduction rate of syndrome scores. The improvement effect of BYQTC in COPD patients was evaluated using forced expiratory volume in 1 s, 6-minute walk test, modified British Medical Research Council and COPD Assessment Test. The level of serum interleukin 8 (IL-8) and matrix metalloproteinase 9 (MMP-9) was evaluated by enzyme-linked immunosorbent assay.

**Results:**

The observation group had a higher clinical control and effectual rate than the control group. After treatment, the improvement of pulmonary function and movement endurance in the observation group was stronger than that in the control group. Meanwhile, the clinical symptoms of COPD were alleviated more in the observation group. Additionally, the decrease in serum IL-8 and MMP-9 levels in the observation group was higher than that in the control group. Finally, the acute exacerbation and rehospitalization of the COPD patient in the observation group was less than that in the control group.

**Conclusion:**

BYQTC significantly enhances the efficacy of conventional therapy in stable COPD patients, resulting in improvements in clinical symptoms, lung function, and overall quality of life. Notable enhancements were observed in FEV1%, 6MWT distance, and reductions in mMRC and CAT scores. Additionally, BYQTC treatment led to substantial decreases in IL-8 and MMP-9 levels, and reduced the incidence of acute exacerbations and rehospitalizations, demonstrating its superior therapeutic benefit compared to standard treatment.

## Introduction

Chronic obstructive pulmonary disease (COPD) is a common respiratory disease with an incidence rate, rapid progress, disability rate, and mortality rate (1, 2). It was reported that the morbidity of COPD in over 40-year-old adults ranged from 5 to 19% (3), and it will be the third global leading cause of death by 2030 (4). COPD has become one of the most prevalent chronic illnesses in China, with around 100 million patients and an 8.6% morbidity rate in over 20-year-old adults (5). Chronic inflammation and irreversible airflow obstruction are the main characteristics of COPD, often accompanied by structural changes in the lung (6, 7). Therefore, it is necessary to strengthen early effective treatment interventions for patients and improve their prognosis.

The specific mechanism of COPD is not profoundly understood and specific treatment methods in clinical practice are not sufficient. Presently, most clinical treatment drugs including bronchodilators, antioxidants, expectorants, antibiotics, phosphodiesterase inhibitors, and glucocorticoids are applied for patients with COPD (8). The above drugs can improve the clinical symptoms of patients and control the disease progression; however, patients under treatment for a long time are more likely to develop intestinal complications, such as functional disorders, osteoporosis, and fungal infections. Meanwhile, the high treatment cost, the poor treatment compliance of patients, the recurrence of disease, and the poor treatment effect hinder its wide application in clinics (9). Traditional Chinese medicine (TCM) has long been used for the clinical treatment of COPD (10). Statistical analyses showed that the number of randomized control trials related to the treatment of COPD with exclusive TCM has been increasing (11). TCM should be considered as part of the therapeutics schedule for COPD patients because it may regulate immune imbalance, protease-antiprotease imbalance, pathological changes in lung function, improve airway remodeling, control lung inflammation, and oxidative-antioxidative processes (12). Wang et al. (13) demonstrated that the TCM formula was better than conventional Western medicine alone in enhancing lung function, improving the quality of life in COPD patients, alleviating clinical symptoms, and exhibiting a more favorable safety profile.

The clinical manifestations of COPD include wheezing and shortness of breath, coughing and phlegm, chest fullness, chest tightness and congestion, or cyanosis of labial armor, palpitations and swelling, and other related symptoms. Therefore, based on the clinical syndrome characteristics of COPD, it is classified under the traditional Chinese medicine (TCM) categories of “coughing disorders, wheezing disorders, and lung distention.” It is believed that the etiology and pathogenesis of COPD are characterized by lung and kidney deficiency as the root cause, and phlegm stasis and coagulation as the basis for the formation of standard excess. In the treatment of stable COPD, both symptomatic relief and addressing the underlying causes are emphasized. Therefore, the therapeutic principle of “tonifying the lungs and benefiting the kidneys, dispelling phlegm and unblocking collaterals” has been established for COPD management. Based on this treatment principle, Bufei Yishen and Qutan Tongluo Cream (BYQTC) were prescribed. The objective of this study is to evaluate the clinical efficacy of BYQTC and its effect on the improvement of clinical symptoms in stable COPD patients.

## Methods and materials

### The sample size estimation

The formula used to calculate the sample size was *n* = (*Z*_*α*/2_ × *σ*/*E*)^2^, where n represents the sample size, *Z*_*α*/2_ is the *Z*-value corresponding to the desired confidence level, *σ* denotes the standard deviation, and *E* is the margin of error. The effect size and standard deviation were derived from prior research and existing literature. Assuming *α* = 0.05 and *β* = 0.2, the minimum required sample size for each group was calculated to be 37, totaling 74 participants. To account for potential dropouts, a final total of 96 participants was included in the study.

### The diagnosis of stable COPD patients

All patients who met the following criteria were eligible for the study. Initially, meeting diagnostic criteria for stable COPD as outlined in the 2013 revised version of the Diagnosis and Treatment Guidelines for COPD developed by the Respiratory Branch of the Chinese Medical Association. Secondly, meeting the diagnostic criteria for pulmonary distension disease (lung kidney Qi deficiency combined with phlegm turbidity obstructing lung syndrome) in the Chinese Medicine Internal Medicine (10th edition, 2017). Thirdly, lung function with grades II and III. Moreover, the age of patients ranging from 40 to 85 years old. After meeting the inclusion criteria, patients were randomly assigned to the control or observation group using a random number table, without stratification or matching. The study was approved by the Ethics Committee of Hebei Provincial Hospital of Traditional Chinese Medicine. Informed written consent was obtained from the patients. The patient confidentiality and the data were strictly protected.

### The exclusion criteria for COPD patients

Patients would be excluded if they met the following criteria: (1) incomplete clinical data; (2) acute exacerbation period of COPD; (3) concomitant liver disease, urinary system disease, hematological system disease, tumor; (4) accompanied by respiratory diseases such as asthma, interstitial lung disease, pneumonia, pulmonary tuberculosis, lung cancer, pulmonary embolism and so on; (5) suffering from end-stage diseases such as heart failure, acute myocardial infarction, acute cerebral infarction, acute cerebral hemorrhage and so on.

Meanwhile, during the study, those patients also would be excluded from these studies if they met the following criteria: (1) poor compliance and compatibility, including irregular medication use, discontinuation of medication, or use of other medications that may affect the final efficacy assessment; (2) subjects experience an acute exacerbation of COPD during the clinical study period; (3) patients who interrupt this experiment themselves.

### The prescription of BYQTC

The prescription for BYQTC is as followings: 30 g Chenpi, 24 g Qingpinxia, 30 g Poria, 30 g Suzi, 20 g Ephedra, 30 g Almond, 15 g Glycyrrhiza, 30 g Dilong, 30 g *Bombyx batryticatus*, 30 g *Magnolia officinalis*, 30 g Mulberry white skin, 30 g Huajuhong, 40 g Astragalus, 30 g Huangjing, 30 g Baizhu, 30 g Dangshen, 50 g Delicacy, 40 g Chinese yam, 30 g *Psoralea corylifolia*, 20 g Schisandra, 30 g *Fritillaria thunbergia*, 30 g *Anemarrhena asphodeloides*, 30 g Red Peony, 30 g Danshen, 30 g Baibu, 30 g Stir fried Malt, 500 g Yellow Wine, 450 g ass hide glue, 500 Xylitol. A total of 600 mL ointment was made according to the prescription. The 600 mL ointment was divided into 60 packets of 10 mL each. Then, after the ointment has cooled, they were stored it in a clean, dry container. The ointment was heated to soften it before taking it. One packet was taken twice daily, and the ointment was prepared monthly. Each treatment course lasted 60 days. The ointment was made in the decoction room of Hebei Provincial Hospital of Traditional Chinese Medicine. BYQTC was administered orally.

### The treatment approach for enrolled patients

A total of 145 stable COPD patients were evaluated in the Respiratory Department of Hebei Traditional Chinese Medicine Hospital. A total of 96 cases were included in the treatment based on the established inclusion and exclusion criteria. Subsequently, the participants were randomly divided into two groups: a control group and an observation group, with 48 subjects in each group. The COPD patients in the control group were treated with 2.5 μg tiotropium bromide spray (TBS) one time daily. The COPD patients in the observation group were administered with TBS and BYQTC. The therapeutic effect is consequently observed. Then follow-up of 6 months was conducted to monitor the incidence of the acute exacerbation and rehospitalization rates among patients.

### Pulmonary function grading

Pulmonary function classification was conducted in accordance with the relevant standards of the Chinese expert consensus on the diagnosis and treatment of acute exacerbation of COPD. Airflow limitation is defined when the patient’s forced expiratory volume in the first second (FEV1)/forced vital capacity (FEV1%) is less than 70% following the administration of bronchodilators. Pulmonary function is classified into 4 levels based on its severity. Grade I is mild with FEV1% ≥80%, grade II is moderate with FEV1% ranging from 50 to 79%, grade III is severe with FEV1% ranging from 30 to 50%, grade IV is extremely severe with FEV1 <30%. FEV1 and FVC were measured using the Master Scope pulmonary function instrument (Jaeger, Germany).

### TCM syndrome score and efficacy evaluation

According to the “Diagnosis Criteria for Traditional Chinese Medicine Syndrome of Chronic Obstructive Pulmonary Disease (2011 Edition)” and the “Guiding Principles for Clinical Research of New Chinese Medicines,” scoring criteria are developed for symptoms including cough, phlegm, wheezing, fatigue, dyspnea, dyspnea, abdominal distension, loose stools, and spontaneous sweating. The TCM syndrome score of COPD patients was assessed both pre- and post-treatment, and the improvement rate of the TCM syndrome score was calculated using the Nimodipine method. The reduction rate of the syndrome score = (Pre-treatment syndrome score − Post-treatment syndrome)/Pre-treatment syndrome score × 100%.

The clinical efficacy was divided into clinical control, effectual, effective, and ineffective according to the reduction rate of the syndrome score. When the clinical symptoms disappeared or basically disappeared, and the reduction rate of the syndrome score is greater than or equal to 95%, it was defined as clinical control. When the clinical symptoms were significantly improved, and the reduction rate of the symptoms score ranged from 70 to 95%, it was defined as effectual. When the clinical symptoms were improved, and the reduction rate of the symptoms score ranged from 30 to 70%, it was defined as effective. There is no significant improvement or even aggravation in clinical symptoms, and the reduction rate of the syndrome score is less than 30%, it was defined as ineffective.

### 6-minute walk test

This test refers to the 2002 American Thoracic Association’s 6-minute Walk Guide. Before the experiment, the purpose and key points of the operation were introduced to the patients, and the examiner could provide a demonstration if necessary. A straight corridor was chosen as the testing site to ensure smooth and unobstructed conditions, where two cone-shaped traffic signs were marked as starting and turning points. Afterwards, the patient was told to walk as much as possible within 6 min under the permit of their conditions while running or jogging is not allowed. During the experiment, encourage patients appropriately and record the distance they can walk within 6 minute.

### COPD Assessment Test scoring

COPD Assessment Test (CAT) scoring has eight clinical indexes, where each clinical index having 6 points from “0” to “5.” Not coughing at all is considered a “0,” and always coughing is considered a “5.” The remaining clinical indexes include no sputum, no chest tightness, difficulty breathing caused by climbing one level of stairs, no household chores affected by COPD, going out anytime, good sleep and intense energy.

### Modified Medical Research Council Dyspnea Scale

The modified Medical Research Council Dyspnea Scale (mMRC) includes five grades. The patient only experiences difficulty breathing during strenuous exercise, with a score of 0. The patient experiences shortness of breath while briskly walking on flat ground or climbing small slopes, with a score of 1. The patient walks on flat ground slower than peers or needs to stop to rest due to shortness of breath, with a score of 2. The patient walks about 100 meters on flat ground or needs to stop to catch my breath, with a score of 3. The patient is unable to leave home due to severe breathing difficulties or has difficulty breathing while putting on or taking off clothes, with a score of 4.

### The IL-8 and MMP-9 detection

On the day before treatment and 3 months after treatment, 3 mL of elbow vein blood was collected from the antecubital vein of COPD patients after an overnight fast in the morning. The serum was separated by centrifugation for 10 min. Enzyme-linked immunosorbent assay was used to measure the levels of interleukin 8 (IL-8), and matrix metalloproteinase 9 (MMP-9) with a commercial kit (Abcam, Cambridge, MA, United States).

### Statistical analysis

All statistical analysis was performed using SPSS software, version 26.0. The statistical data was as shown mean ± SD. *p*-values less than 0.05 indicated the significance of the test. The Shapiro–Wilk test was used to assess the normality of the data before analysis. The comparison of the basic information was done by the Mann–Whitney test or Fisher’s exact test. The comparison of treatment effect in two groups was conducted using ANOVA followed by Tukey’s multiple comparisons tests.

## Results

### Research flow of this study

**Figure 1:**
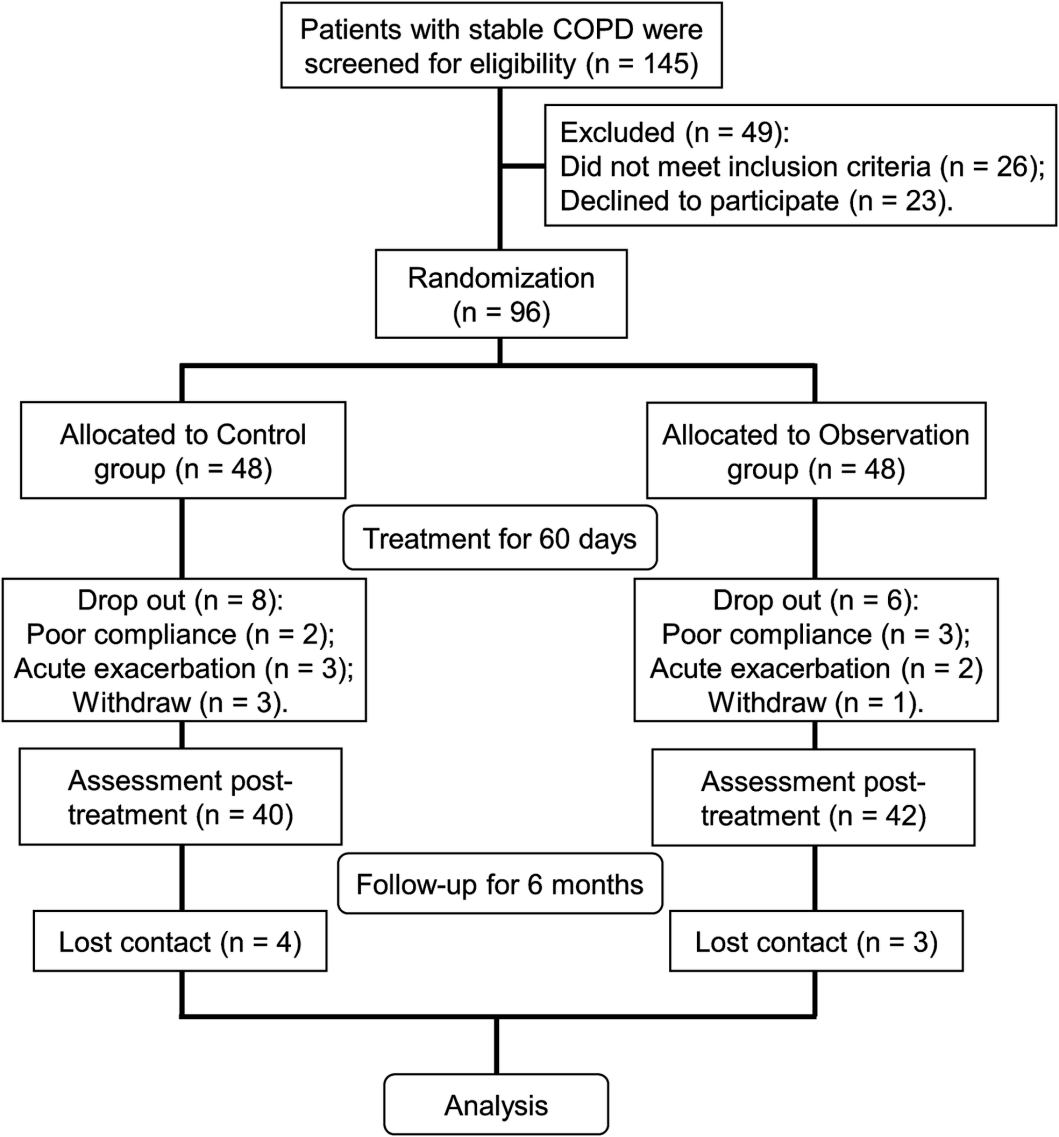
Research flow of this study.

A total of 145 stable COPD patients were evaluated in the Respiratory Department of Hebei Traditional Chinese Medicine Hospital. According to the inclusion and exclusion criteria, 96 patients were enrolled for further study. Ninety-six cases were randomly divided into a control group and an observation group using a random number table (Figure 1). The control group was treated with 2.5 μg TBS one time daily. The observation group was treated with TBS in combination with BYQTC (Figure 1). The treatment group and the control group received 60 days of treatment as one course, after which the therapeutic outcomes were assessed and documented (Figure 1). Consequently, follow-up of 6 months was conducted to monitor the patient’s acute exacerbation and rehospitalization status. During the treatment period, eight cases in the control group and six cases in the observation group were lost to follow-up (Figure 1). Herein, 40 cases from the control group and 42 cases from the observation group were included in the final analysis. During the six-month follow-up period after treatment, four patients in the control group and three patients in the observation group were lost to follow-up (Figure 1).

### The main characteristics of enrolled stable COPD patients

The clinical characteristics of the COPD patients in two groups was compared. It was found that the age and gender distribution have no difference between the two groups (Table 1). Afterwards, the disease history of the COPD patients was also evaluated and the results revealed that the percentage of smoking history, hypertension, coronary heart disease, and diabetes mellitus in COPD patients among the two groups was similar (Table 1). Importantly, the COPD course and the pulmonary function grade of the COPD patients among the two groups were comparable (Table 1).

**Table 1:** Baseline characteristics of stable chronic obstructive pulmonary disease (COPD) patients who received the treatments of tiotropium bromide spray (control) or tiotropium bromide spray combined with Chinese medicine (observation).

Characteristics	Control (*n* = 40)	Observation (*n* = 42)	*p*-value
Age (years)	62.37 ± 9.91	63.05 ± 10.58	0.274
COPD course (years)	10.42 ± 3.17	10.84 ± 3.69	0.382
Gender			
Male	33 (82.5%)	30 (71.4%)	0.299
Female	7 (17.5%)	12 (28.6%)	
Smoke history			
Yes	29 (72.5%)	35 (83.3%)	0.291
No	11 (27.5%)	7 (16.7%)	
GOLD			
II	17 (42.5%)	14 (33.3%)	0.495
III	23 (57.5%)	28 (66.7%)	
Hypertension			
Yes	15 (37.5%)	13 (30.9%)	0.643
No	25 (62.5%)	29 (69.1%)	
Diabetes mellitus			
Yes	6 (15%)	10 (23.8%)	0.407
No	34 (85%)	32 (76.2%)	
Coronary heart disease			
Yes	5 (12.5%)	4 (9.5%)	0.735
No	35 (87.5%)	38 (90.5%)	

The data are presented as mean ± SD or *n* (percentage). The comparisons of data were done by Mann–Whitney test or Fisher’s exact test. GOLD, Global Initiative for Chronic Obstructive Lung Disease.

### Comparison of clinical effectiveness of stable COPD patients

The improvement rate of the TCM syndrome score was used to evaluate the clinical effect. The analysis result showed that the clinical control rate is 7% in the observation group and 2% in the control group; the effectual effect is 23% in the observation group and 13% in the control group. However, the effective and ineffective rates in the observation group decreased compared to the control group (Table 2, *p* = 0.018). Collectively, the clinical effect of the observation group was better than that of the control group.

**Table 2:** Comparison of clinical effectiveness of stable chronic obstructive pulmonary disease patients (COPD) patients who received the treatments of tiotropium bromide spray (control) or tiotropium bromide spray combined with Chinese medicine (observation).

	Study group		*p*-value
	Control (*n* = 40)	Observation (*n* = 42)	
Clinical control	2 (%)	7 (%)	0.018
Effectual	13 (%)	23 (%)	
Effective	16 (%)	8 (%)	
Ineffective	9 (%)	4 (%)	

Values were expressed as *n* (percentage, %). *p*-value was derived from Chi-square test.

### The improvement assessment of COPD patients after therapeutics

**Figure 2:**
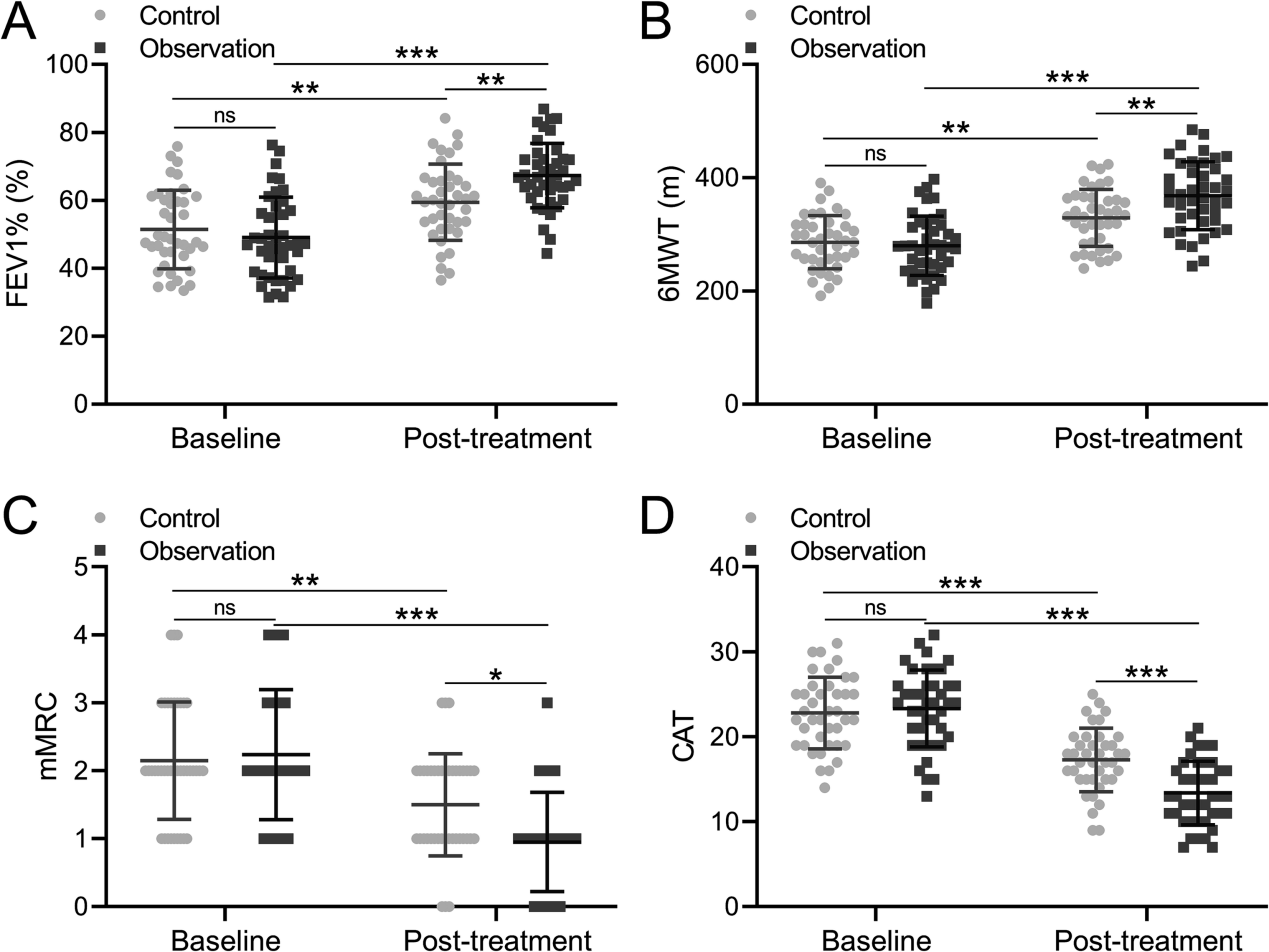
Comparisons of FEV1% **(A)**, 6MWT **(B)**, mMRC **(C)** and CAT **(D)** before and after the treatment between patients who received the treatments of TBS (control, *n* = 40) or TBS combined with Chinese medicine (observation, *n* = 42). The data are presented as mean ± SD. ^*^p < 0.05, ^**^p < 0.01, and ^***^p < 0.001 and ns means no significance from mix model of ANOVA followed by Tukey’s multiple comparisons tests.

FEV1% is a global measurement of lung function (14). Our analysis unveiled that FEV1% was 51.414% in the control group and 49.028% in the observation group respectively, which are comparable. After treatment, the FEV1% was increased in the control group at 59.416 percent and also increased in the observation group at 67.283 percent, which indicated that the observation group had a higher increase of FEV1% compared to the control group (Figure 2A, *p* = 0.008).

The 6-minute walk test (6MWT) is a widely used assessment method for walking endurance in sub-maximal conditions (15). Subsequently, the 6-minute walk test (6MWT) was conducted for COPD patients, with results indicating that the 6MWT were 286.000 meters and 279.729 meters in the two groups, demonstrating comparable outcomes. After treatment, the distances between the two groups were increased to 328.99 meters in the control group and 368.093 meters in the observation group, which implied that the increment of 6MWT in the observation group was greater than that in the control group (Figure 2B, *p* = 0.005).

MMRC is a clinical score used to evaluate the severity of respiratory distress in patients. The CAT score systematically and comprehensively quantified the life quality of patients affected by COPD from the main symptom score and life state score. Global Initiative for Chronic Obstructive Pulmonary Disease (GOLD) recommends the CAT or the mMRC dyspnea scale to assess symptoms in patients with COPD. Consequently, the mMRC and CAT were applied to evaluate the COPD patients. The analysis showed that the mMRC was 2.15 in the control group and 2.238 in the observation group before treatment, while mMRC was 1.5 in the control group and 0.952 in the observation group after treatment. The result demonstrated that compared to the control group, the observation group showed a greater decrease in mMRC (Figure 2C, *p* = 0.017). Similarly, the CAT was 22.800 in the control group and 23.333 in the observation group before treatment, while it was 17.275 in the control group and 13.381 in the observation group after treatment, which proved that the observation group showed a greater decrease in CAT compared to the control group (Figure 2D, *p* < 0.001). Taken together, it was concluded that the COPD-related symptoms can be effectively improved, meanwhile, the clinical effect of combination treatment with BYQTC was better than traditional treatment.

### The assessment of IL-8 and MMP-9

**Figure 3:**
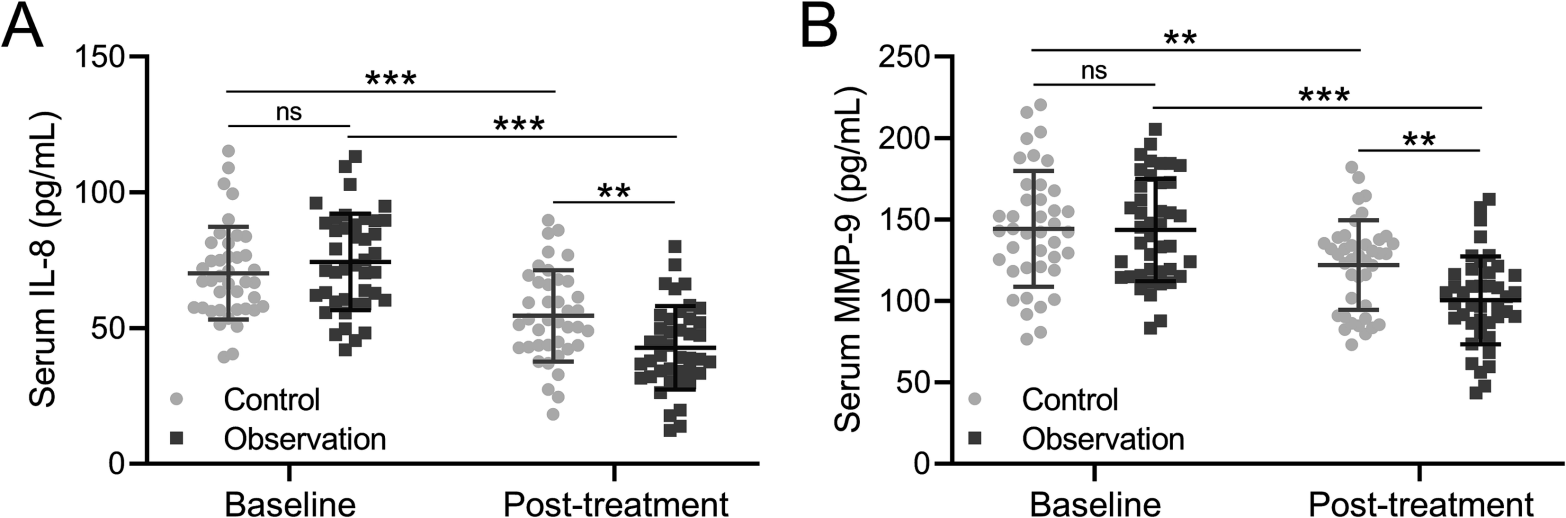
Comparisons of serum IL-8 **(A)** and MMP-9 **(B)** before and after the treatment between patients who received the treatments of TBS (control, *n* = 40) or TBS combined with Chinese medicine (observation, *n* = 42). The data are presented as mean ± SD. ^**^p < 0.01 and ^***^p < 0.001 and ns means no significance from the mixed model of ANOVA followed by Tukey’s multiple comparisons tests.

IL-8 plays an important role in the inflammatory response of COPD airways. IL-8 can induce infiltration of neutrophils, eosinophils, and monocytes, and macrophages present in the airway mucosa. Therefore, the level of IL-8 in COPD patients was detected before and after treatment. The analysis result showed that the level of IL-8 was comparable in the control and observation groups with 70.231 pg/mL and 74.344 pg/mL before treatment (Figure 3A). After treatment, the level of IL-8 was decreased to 54.515 pg/mL in the control group while 42.727 pg/mL in the observation group (Figure 3A, *p* = 0.009), showing that the decrease of IL-8 level was greater in the observation group than the control group. MMP-9 is involved in the entire process of COPD and plays an important role in the occurrence and development of systemic inflammation and airway inflammation. The level of MMP was 144.332 pg/mL in the control group and 143.574 pg/mL, while it decreased to 122.015 pg/mL in the control group and 100.385 pg/mL in the observation group, which proved that the MMP-9 level decreased greater in the observation group (Figure 3B, *p* = 0.008). Based on these findings, it was concluded that these two factors in COPD patients’ serum were effectively improved after treatment. However, the effect of BYQTC as an adjuvant treatment was significantly better than traditional treatment.

### The follow-up of the COPD patients after treatment

**Figure 4:**
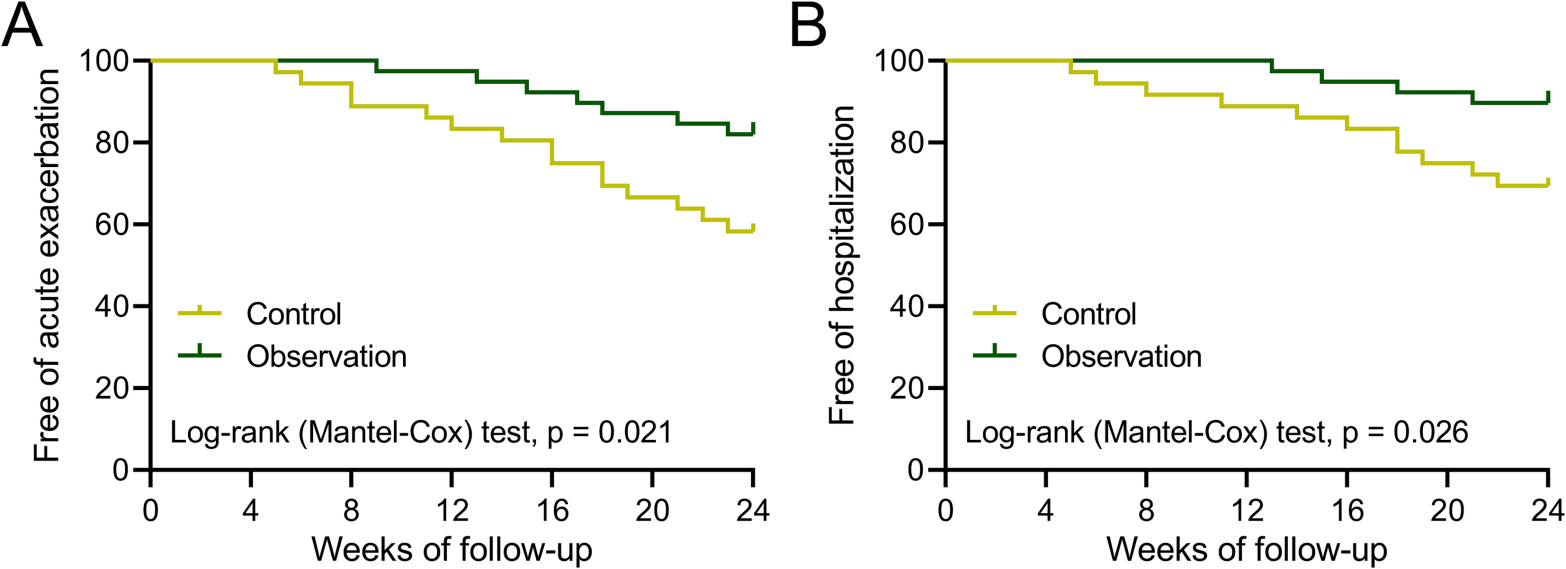
Curves of free of acute exacerbation **(A)** and hospitalization **(B)** during the 24 weeks of follow-up after treatment between patients who received the treatments of TBS (control, *n* = 36) or TBS combined with Chinese medicine (observation, *n* = 39).

During the six-month follow-up period post treatment, four and three patients in both groups were out of contact. Therefore, the free of acute exacerbation and rehospitalization were analyzed among 36 patients in the control group and 39 patients in the observation group. The analysis result showed that the percentage of free acute exacerbation in the observation group was higher than that in the control group (Figure 4A, *p* = 0.021). Meanwhile, the percentage of free rehospitalization in the observation group was also higher than that in the control group (Figure 4B, *p* = 0.026). These findings demonstrated that the adjuvant therapy of BYQTC had a better effect on decreasing the incidence of acute exacerbation or rehospitalization of COPD patients within 6 months after treatment with conventional treatment.

## Discussion

COPD is a serious global health burden, with a high prevalence affecting approximately 10% of the adult population and an increasing incidence. Recently, the morbidity and mortality of COPD are still high, which has brought high hospitalization costs and drug costs to families and society in urban or rural areas of China. Due to its growing incidence, the World Health Organization and the US National Institute of Health experts established the GOLD and outlined the Global Strategy for COPD Diagnosis, Management, and Prevention (16). Therefore, finding more effective clinical treatment methods for COPD is of great significance in improving prognosis, reducing the risk of death, enhancing patient labor and quality of life, and reducing the social and economic burden caused by the disease. Compared with patients in the acute exacerbation phase, patients in the stable phase have relatively mild clinical symptoms and stable conditions (17). The management of stable COPD has always been a focus of domestic and foreign guidelines and scholars. The GOLD 2020 guidelines emphasize that the primary goals of managing stable COPD are to alleviate symptoms, improve exercise endurance, improve health status, and most importantly, reduce the risk of deterioration (18).

A recent epidemiological study showed that the incidence of COPD increased with age in China (19). In terms of the gender difference, the incidence of COPD was higher in males than in females, which may be attributed to the higher prevalence of smoking among males (19). Therefore, the enrolled COPD patients in the control group and observation group have no difference in age and gender distribution, which excludes risk factors that affect the effect of treatment. Moreover, almost all available data has demonstrated that the incidence of COPD in the smokers population was higher than that in the non-smokers population (20). It is estimated that 28.1% of adults aged over 15 years are smokers, accounting for approximately 1/3 of the global smoking population (21). So, the percentage of the smoking population in the control group and observation group was similar, thereby mitigating the potential confounding effect of smoking on the treatment outcomes. Additionally, pulmonary hypertension is a common complication of advanced COPD and is defined by mean pulmonary artery pressure (22). Meanwhile, it was also evidenced that COPD was associated with diabetes (23). Interestingly, it was also proved that COPD was a risk factor for coronary heart disease (24). Taken together, the percentage of COPD patients with hypertension, diabetes, or coronary heart disease was similar in the control and observation groups.

Presently, the clinical therapeutic approaches for stable COPD mainly include respiratory training and drug intervention. Resistance breathing training is the main method for patients to perform lip and abdominal breathing, which can improve respiratory muscle strength (25). Tiotropium is a long-acting anticholinergic drug used to dilate the clinical bronchi. It can selectively bind M1 and M3 receptors, thereby acting as a certain inhibitory effect on airway inflammation and enhancing lung function in patients (26, 27). Continuous treatment with tiotropium significantly lowers the number of exacerbations and the risk of hospitalization due to exacerbations compared to placebo (28, 29). However, it has a high cost for COPD patients.

Research has shown that TCM for stable COPD treatment possessed the characteristics of multiple targets and pathways, which could effectively delay disease progression, alleviate clinical symptoms, and improve quality of life (30, 31). Based on the clinical characteristics of COPD with lung and kidney deficiency syndrome, BYQTC was developed as a therapeutic formulation. This prescription consisted of Jun drugs, Chen drugs, and Shi Drugs. Jun Drugs include *Astragalus membranaceus*, *Rehmannia glutinosa*, Huangjing, Ziheche, and Deer antler gum, which could achieve warming, tonifying the lungs, and strengthening the kidneys. Chen drugs consist of dried ginger, codonopsis pilosula, *Atractylodes macrocephala*, Chenpi, Qingbanxia, and yam, which are used to warm the yang, strengthen the spleen, remove dampness, and transform the drink; Earthly dragon and *Salvia miltiorrhiza* which are used to promote blood circulation, remove blood stasis, and unblock collaterals; Asarum, *Schisandra chinensis*, and Buguzhi, which are used to tonify the lungs and kidneys, nourish qi and relieve asthma. Shi drugs include Mulberry bark with functions of clearing heat and promoting lung function, while honey with functions of relieving cough, resolving phlegm, and harmonizing various medicines. The whole formula is effective in tonifying the lungs, tonifying the kidneys, removing phlegm, and unblocking the meridians. Our study showed that the clinical control rate of COPD patients treated with TBS and BYQTC was higher than those treated with TBS alone. It indicated that BYQTC could enhance the effect of TBS on COPD. Importantly, the assessment of COPD using mMRC and CAT revealed that the clinical symptoms of the COPD patients treated with the combined approach were more significantly alleviated compared to those treated with TBS alone. Additionally, pulmonary function and movement ability were improved to a greater extent in COPD patients receiving the combined treatment than in those treated with TBS alone.

The dominant pathological and pathophysiological changes of COPD are progressive airflow limitation and peripheral airway inflammation (32). It was found that IL-8 was significantly elevated in stable COPD (33). Meanwhile, it has been reported that the mRNA levels of MMP-9 were more than two-fold higher in severe COPD patients compared to non-COPD smokers or those with moderate COPD, indicating that MMP-9 serves as a biomarker for the grade and severity of COPD (34). Our result showed that the level of serum IL-8 and MMP-9 were lower in COPD patients treated with a combination approach than those treated with TBS alone after treatment, which was also consistent with the previous study.

Our results highlighted the potential of integrating BYQTC into long-term COPD management, either as a standalone therapy or in combination with conventional treatments, offering a promising approach to improve patient outcomes. Future research should focus on evaluating BYQTC’s ability to provide sustained symptom relief, reduce exacerbation frequency, and enhance overall quality of life. A comparative analysis of BYQTC’s efficacy with other traditional Chinese medicine therapies and conventional Western treatments, such as bronchodilators and corticosteroids, would provide important insights into optimal treatment strategies. Additionally, assessing the long-term safety profile and cost-effectiveness of BYQTC is crucial for its broader clinical implementation.

Although our study demonstrated that BYQTC could enhance the treatment effect of TBS in the alleviation of clinical symptoms and improvement of lung function, there are still some issues to be addressed in the future. First of all, the single TCM should be taken into consideration that whether single TCM could improve the symptoms of COPD patients. Moreover, the sample size was too limited to obtain a convincing conclusion that TCM could enhance the treatment effect of TBS. Therefore, more COPD patients would be included in the analysis. Furthermore, the open-label design may introduce performance bias, which could be addressed in future randomized double-blind studies. Additionally, the generalizability of these findings is limited by the exclusive inclusion of Chinese participants, as ethnic differences in TCM response and COPD pathophysiology could affect outcomes. Future studies should include multinational cohorts to assess cross-population applicability.

## Data Availability

The original contributions presented in the study are included in the article/supplementary material, further inquiries can be directed to the corresponding authors.
